# Development of Genomic Resources for a thraustochytrid Pathogen and Investigation of Temperature Influences on Gene Expression

**DOI:** 10.1371/journal.pone.0074196

**Published:** 2013-09-17

**Authors:** Ana Elisa Garcia-Vedrenne, Maya Groner, Annie Page-Karjian, Gregor-Fausto Siegmund, Sonia Singhal, Jamie Sziklay, Steven Roberts

**Affiliations:** 1 Department of Ecology, Evolution & Marine Biology, University of California, Santa Barbara, Santa Barbara, California, United States of America; 2 Department of Health Management, Centre for Veterinary Epidemiological Research, University of Prince Edward Island, Charlottetown, Canada; 3 Department of Pathology, College of Veterinary Medicine, University of Georgia, Athens, Georgia, United States of America; 4 Department of Ecology and Evolution, University of Chicago, Chicago, Illinois, United States of America; 5 Department of Biology, University of Washington, Seattle, Washington, United States of America; 6 Hawai’i Institute of Marine Biology, University of Hawai‘i, Kane‘ohe, Hawai‘i, United States of America; 7 School of Aquatic & Fishery Sciences, University of Washington, Seattle, Washington, United States of America; University of Pittsburgh, United States of America

## Abstract

Understanding how environmental changes influence the pathogenicity and virulence of infectious agents is critical for predicting epidemiological patterns of disease. Thraustochytrids, part of the larger taxonomic class Labyrinthulomycetes, contain several highly pathogenic species, including the hard clam pathogen quahog parasite unknown (QPX). QPX has been associated with large-scale mortality events along the northeastern coast of North America. Growth and physiology of QPX is temperature-dependent, and changes in local temperature profiles influence pathogenicity. In this study we characterize the partial genome of QPX and examine the influence of temperature on gene expression. Genes involved in several biological processes are differentially expressed upon temperature change, including those associated with altered growth and metabolism and virulence. The genomic and transcriptomic resources developed in this study provide a foundation for better understanding virulence, pathogenicity and life history of thraustochytrid pathogens.

## Introduction

Quahog Parasite Unknown (QPX) is a thraustochytrid protistan parasite that has been implicated as the causative agent of large-scale mortalities in hatchery-reared and commercially harvested hard clams (quahogs; *Mercenaria* *mercenaria* ) throughout the northeastern coast of North America (Prince Edward Island, Canada, and Virginia, Massachusetts, New York, and New Jersey, USA) [1-7]. Outbreaks remain a concern within the shellfish industry, since several naïve fisheries exist within the QPX range (Prince Edward Island, Canada to Virginia, USA) [8]. QPX lies within the larger taxonomic class of Labyrinthulomycetes, a poorly described protistan fungal-like group consisting of saprophytes and some parasites, several of which are highly pathogenic [9-12]. Development of genomic resources for QPX will provide important insight into the physiology of this pathogen and generate a foundation for a future phylogenetic placement within the labyrinthulomycetes.

As is the case with many marine pathogens [13], temperature plays an important role in QPX disease dynamics. In sites where QPX is found, sea surface temperatures range from -1.2°C to 2°C in the winter and 17°C to 27°C in the summer [14,15]. Several *in vitro* studies have demonstrated that QPX growth is greatest in standard culture medium between 20°C and 23°C [16]. Similarly, *in vitro* studies show that mucus production is temperature dependent and is greatest at 24°C, compared to production at 0°C, 16°C, and > 32°C [13]. Mucus production by QPX is considered a primary virulence mechanism, with mucus secretion promoting clam inflammatory responses via chemoattractant activity [17] while also providing the pathogen protection from clam phagocytic hemocytes [6] and humoral antimicrobial agents [18]. The majority of mortality events in the field occur during the summer and early fall [1,3,6]. Interestingly, *in vivo* infection trials showed higher disease prevalence and intensity in naturally infected clams at lower temperatures (13°C compared to 21°C and 27°C) [19]. The apparent discrepancy between higher disease prevalence and reduced host healing at cooler temperatures and mortality in the summer has been attributed to the fact that QPX is a chronic disease, and summer mortality is viewed as an endpoint of an ongoing infectious process [4]. The important role that temperature plays from both the pathogen and host perspectives will continue to garner attention with shifting sea surface temperature gradients.

While numerous studies examine QPX in ecological [5,20] and physiological contexts [13,21], there are limited genomic resources for this species. Recent advances in high-throughput sequencing technologies have facilitated the development of genomic resources that can be instrumental in identifying physiological responses to altered environmental conditions. For instance, RNA-Seq, one of the methods used here, allows for unbiased, high-resolution analysis of global gene expression patterns. The objectives of this study were 1) to generate genomic resources for this pathogen; and 2) to examine the influence of temperature on gene expression patterns in QPX. These data provide important insight into the molecular mechanisms underlying physiological changes associated with temperature. To our knowledge, this is the first report of genomic and transcriptomic characterization in an environmentally and commercially important shellfish pathogen.

## Results

### Genomic Sequence Characterization

Genomic sequence data is available in the NCBI Short Read Archive (Accession # SRX197335). *De novo* assembly of the quality trimmed sequencing reads (110,422,312) from the genomic library resulted in 21,280 contigs with a minimum size of 100 bp; of those contigs, there were 6,848 contigs with a minimum size of 1000 bp and 555 contigs with a minimum size of 10,000 bp (Dataset S1). Additional statistics are provided in Table 1. Single nucleotide polymorphism (SNP) detection analysis identified 25,829 putative SNPs across 7,734 DNA contigs (Table S1). The average occurrence of SNPs is 0.7 SNPs per 1,000 bps.

**Table 1:** Characteristics of QPX genomic assembly.

**Assembly Parameter**	**Value**
Number of contigs	21,280
N50 contig length	5.6 kb
Total contig length	34.7 Mb
Average contig length	1629 bp
G+C content (%)	33.4

### Transcriptome Characterization

**Figure 1:**
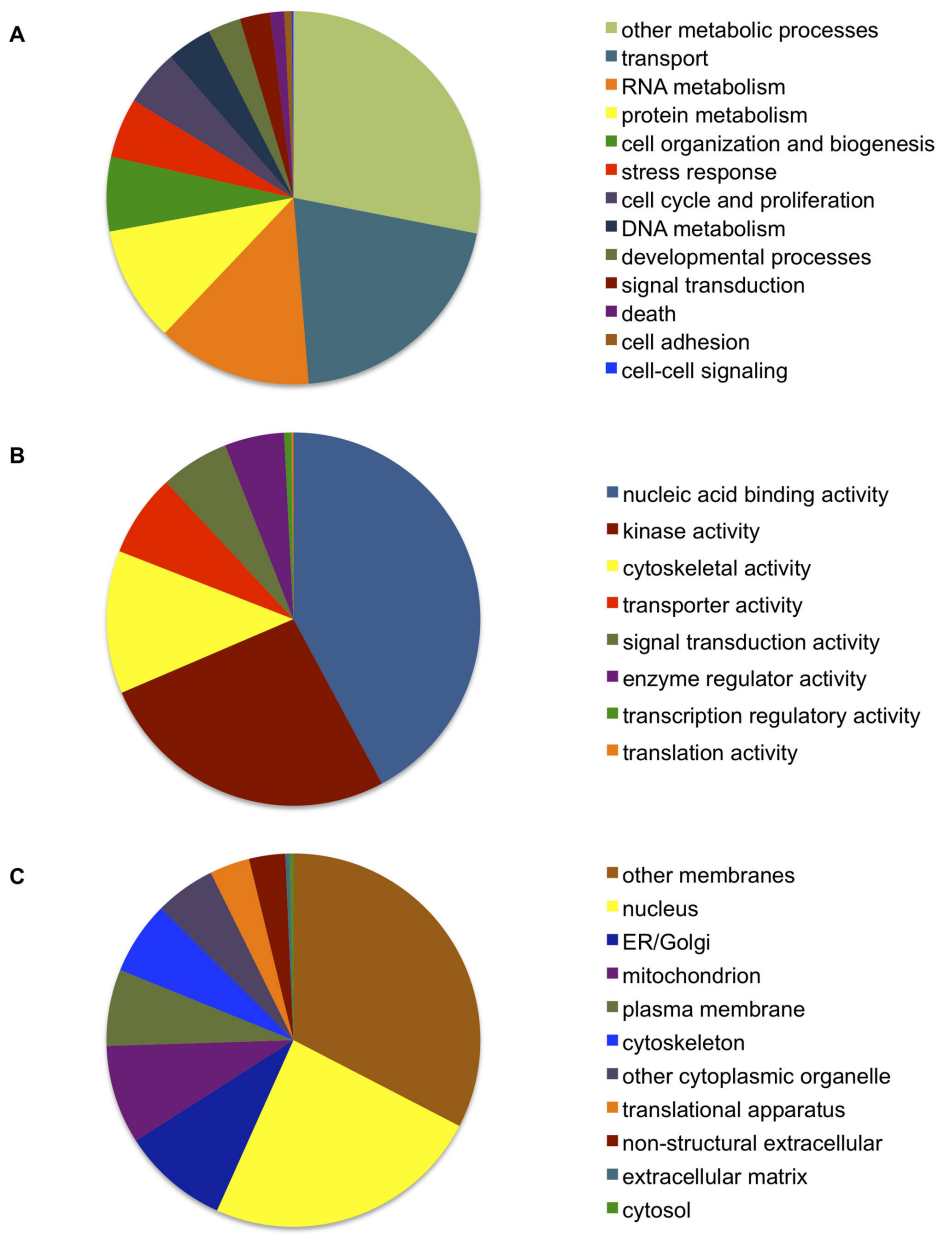
Classification of annotated QPX contigs based on Gene Ontology. Representation of (a) biological processes, (b) molecular function, and (c) cellular components from Gene Ontology Slim terms based on Swiss-Prot gene annotations. The gene ontology categories ‘other biological processes functions’ (a), ‘other molecular functions’ (b), and ‘other cellular components’ (c) were excluded from these graphs.

In addition to examining the pathogen’s partial genome, we characterized the QPX transcriptome. Following quality trimming, 45,082,610 and 30,035,397 reads (36 bp) remained from the transcriptomic libraries generated from QPX incubated at 10°C (QPX10) and at 21°C (QPX21), respectively. Sequence data is available in the NCBI Short Read Archive (Accession # SRX197332). *De novo* assembly of the reads from the two transcriptomic libraries resulted in 59,931,497 reads assembled to produce 11,271 contigs with an average length of 1,176 bp (Dataset S2). Of the 11,271 sequences, 5,992 were annotated using the SwissProt database (Table S2). Corresponding Gene Ontology (GO) classifications for annotated sequences are shown in Figure 1. GO Slim classifications of all annotated contigs are provided in Table S3.

**Figure 2:**
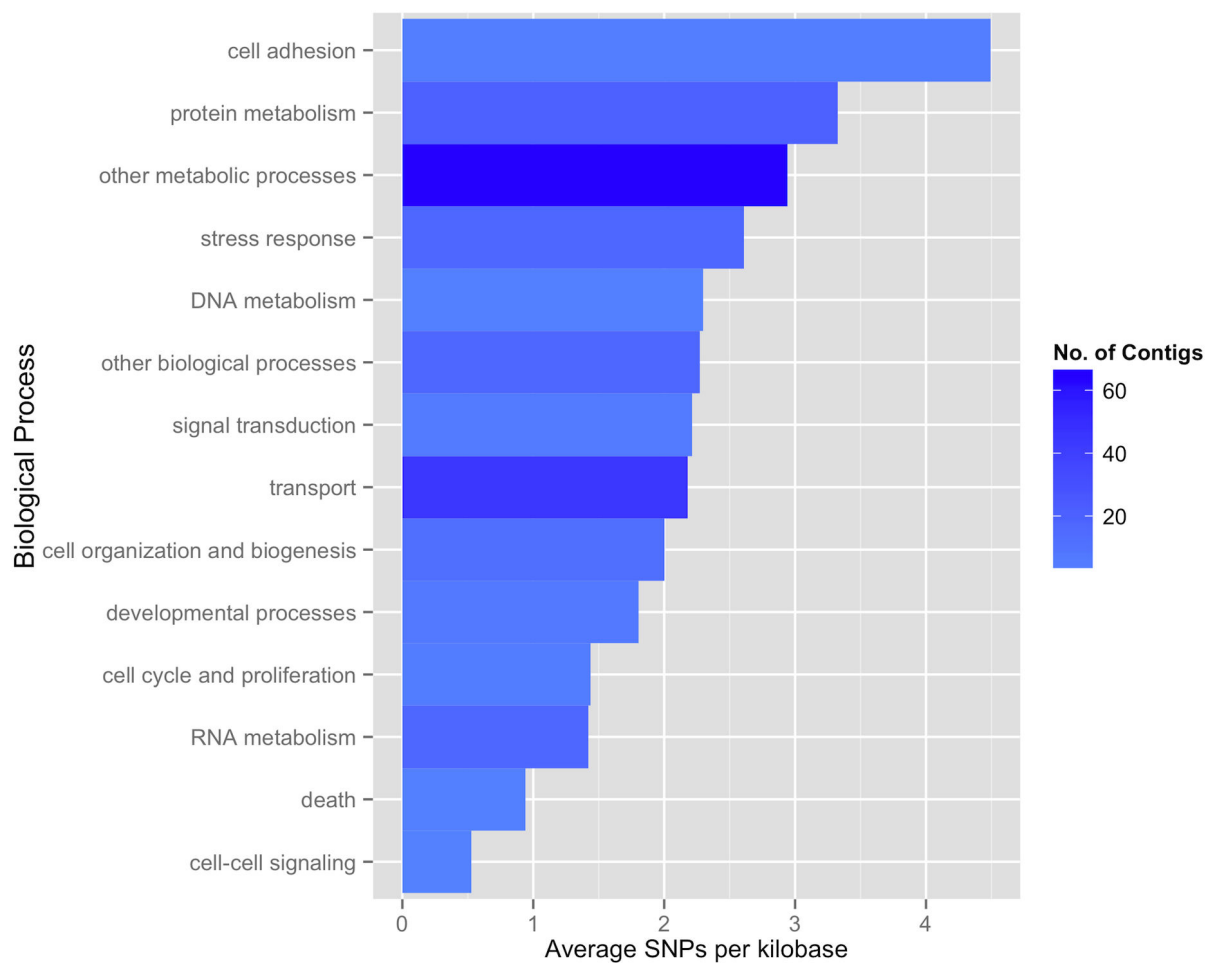
Average number of SNPs per kilobase pair in 152 contigs associated with GO Slim biological processes. Bar heights represent the average SNP rate per kilobase pair for select GO Slim biological processes. Color intensity of the bars indicates number of contigs for each GO Slim term.

There were 1,925 putative SNPs identified from the *de novo* assembly of the transcriptome. These SNPs were identified in 702 contigs, with an average of 3.11 SNPs per contig (of only those containing SNPs) (Table S4). Of the 702 contigs where SNPs were identified, only 152 of the contigs were annotated. We took a closer look at these 152 contigs and characterized the distribution of the 629 putative SNPs identified in these contigs. In the annotated contigs, the average SNP density was higher in genes associated with protein metabolism and cell adhesion (Figure 2).

### RNA-Seq and Gene Enrichment Analysis

**Figure 3:**
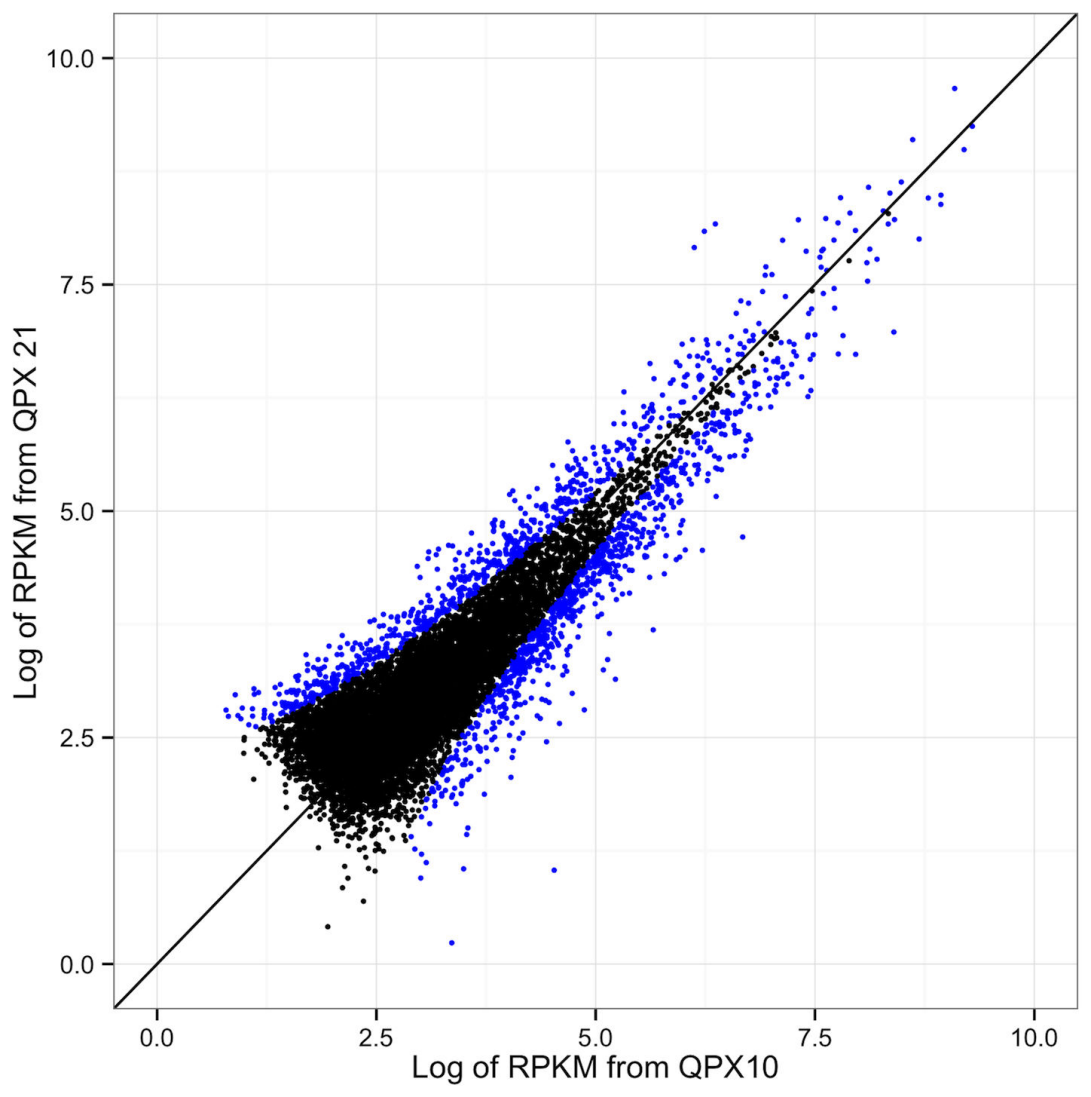
Relative gene expression levels (RPKM) between QPX10 and QPX21 libraries. Each circle represents a single contig, with blue circles indicating those contigs that are differentially expressed. The diagonal line represents equal expression between the two libraries.

Of the 11,271 transcriptome contigs, 849 were expressed at a higher level in the QPX10 library and 728 were expressed at a higher level in the QPX21 library (Figure 3; Table S5). Gene enrichment analysis was performed to evaluate the significance of differentially expressed genes compared to the entire transcriptome. For those genes expressed at an elevated level in the QPX10 library, 26 biological processes were enriched, corresponding to 163 QPX transcriptome contigs (Table S6). For those genes expressed at an elevated level in the QPX21 library, 60 biological processes were enriched, corresponding to 175 QPX transcriptome contigs (Table S6). Enrichment analysis based on gene ontology revealed that enriched biological processes include those associated primarily with translation, response to heat, cellular transport and metabolism.

## Discussion

Several species of labyrinthulids are etiologic agents of disease in aquatic hosts, but little is known about the molecular mechanisms underlying their pathogenicity and virulence [9,11,12,22-25]. The primary aim of this study was to develop genomic and transcriptomic resources to provide tools and information for examining physiological responses, population dynamics and taxonomic relationships for the thraustochytrid pathogen QPX. Prior to this research effort, there were fewer than 2,000 nucleotide sequences available in GenBank [26] for the entire order Labyrinthulidae and fewer than 100 sequences available for QPX. Here we characterize a transcriptome (11,271 contig sequences) and provide a partial genome sequence (34.7 Mb) for this pathogen. Collectively, these data provide information about gene content and expression that will be critical for future studies to identify how physiological responses to stress contribute to temperature-dependent QPX epidemiology.

In order to examine gross genetic variation, putative SNPs were identified in the assembled transcriptome. As expected, we found a relatively high proportion of putative SNPs in genes associated with response to environmental changes (as opposed to those associated with housekeeping functions). Specifically, genes associated with stress response and cell adhesion had higher rates of SNPs per base pair. Genetic variation in cell adhesion is of interest because it has been associated with extracellular mechanisms affecting virulence and host recognition in numerous pathogens, including the protists *Plasmodium malariae* and *Phytophthora* spp. [27]. Some caution should be used in the interpretation of SNPs within the transcriptome; there is likely ascertainment bias due to variation in gene expression, unusual genetic variation resulting from *in vitro* culturing and methodological constraints [28].

In the context of biological processes related to responses to environmental fluctuations, one functional suite of genes identified from our initial transcriptome characterization includes a number of genes associated with responding to stressors. Based on sequence similarity and gene ontology classification, 164 sequences in the QPX transcriptome were identified as being involved in the stress response. These sequences are of interest as stress response encompasses a variety of physiological processes, including those associated with temperature change. Identified sequences include putative orthologs for a phosphatase 2C homolog (Contig_4050) and the ATP-dependent Clp protease ATP-binding subunit ClpC (Contig_55). These proteins are important for regulating growth at elevated temperatures [29], and their identification may yield insight into the variable growth rates of QPX at different temperatures. Genes associated with DNA repair pathways were also identified, including contigs with sequence similarity to nucleotide excision repair (e.g., DNA repair helicase UVH6 (Contig_9981), general transcription factor IIH subunit 4 (Contig_6953); base excision repair (e.g., DNA polymerase lambda (Contig_8122); and mismatch repair (e.g., DNA mismatch repair protein Msh2 (Contig_9728) and DNA mismatch repair protein Mlh1 (Contig_9188)). Interestingly, functional changes in mismatch repair heterodimers have been shown to play an important role in bacterial pathogenesis [30]. The effects of the loss of mismatch repair function are pathogen-specific, and may be beneficial or detrimental [30,31]. Further experiments are required to determine the specific role of DNA repair pathways in QPX, including the mismatch repair pathway.

Another gene of interest identified from our transcriptome sequencing is Thrombospondin-1 (Contig_5706). Thrombospondin-1 forms an extracellular glycoprotein [32], and genes containing repetitive thrombospondin-1 sequences are secreted in pathogenic species of *Phytophthora* spp. during host invasion [27]. Glycoprotein is a primary component of the mucus layer secreted by QPX [33], and the role of the gene in QPX virulence warrants further investigation.

In order to gain a better understanding of the influence of temperature on QPX biology, we performed gene enrichment analysis on differentially expressed genes to identify enriched functional suites of genes. In the remainder of this section we discuss the prominent biological processes found to be enriched based on temperature exposure, including the genes we identified and their putative functions in an ecological context.

As would be expected, one suite of genes found to be influenced by temperature was heat shock proteins (HSPs). Heat shock proteins are involved in the folding and unfolding of proteins and play an integral role in response to temperature and other stressors. The presence of HSPs has been demonstrated in eukaryotes and prokaryotes, and there is evidence that these proteins are crucial for survival of organisms at both normal and altered temperatures [34]. Enriched QPX genes include several encoding small heat shock proteins in the HSP20 family, including 17.6 kDa class I heat shock protein 3 (Contig_186, Contig_101); 18.1 kDa class I heat shock protein (Contig_110); 16.9 kDa class I heat shock protein 3 (Contig_6); and the larger HSP40 chaperone protein DnaJ (Contig_4064, Contig_4637, Contig_275). HSP20 has recently been shown to be associated with motility and host invasion in *Plasmodium malariae* [35] and *Toxoplasma gondii*, and with motility in *Neospora* *caninum* [36]. Interestingly, most of the HSPs that were associated with enriched biological process were expressed at higher levels at 10°C relative to 21°C. One explanation for this pattern is that translational activity is in fact elevated at 21°C relative to 10°C, resulting in the depletion of transcripts at 21°C. In other words, protein expression might be increased at 21°C and an increased rate of translation could deplete the relative transcript abundance. Alternatively, higher transcript levels observed at 10°C could reflect a thermal response in which cooler temperature induced increased gene expression. QPX cultures were maintained at 21°C prior to the experimental trial, and the shift to 10°C could represent an acute environmental stress which triggered a general stress response, as has been observed in yeast [37]. Studies examining proteomic responses would provide important insight into which of the proposed explanations is an accurate description of heat shock protein dynamics in QPX.

A number of genes related to pathogen virulence were identified as contributing to enriched biological processes in the observed differentially expressed genes, particularly a number of proteases. QPX and other thraustochytrids have been shown to extrude several unidentified proteases within a monofilamentous net of mucus, which enables them to break down a large variety of substrates [38,39]. The presence of these proteases suggests a capacity to degrade extracellular matrices of host tissues [38,39], confirmed via histological observation of tissue disruption in QPX-infected clams [1,6]. One gene that was expressed at a higher level at 21°C was beta enolase (Contig_3261). Beta enolase is a glycolytic enzyme that can localize to the cell surface and concentrate plasminogen, a proenzyme of the protein-degrading serine protease plasmin. Enolase production has been suggested as a mechanism of tissue invasion in bacterial and fungal pathogens [40]; thus, it is possible that this transcript represents a component of the mucus secreted by QPX.

Another example of expression of potential factors of QPX virulence at elevated temperature is the upregulation of genes with high sequence similarity to zinc metalloproteinase-disintegrins. Extracellular zinc metalloproteases have been associated with virulence in numerous bacterial and fungal pathogens [41,42]. At 21°C, zinc metalloproteinase-disintegrin jerdonitin (Contig_1521) and zinc metalloproteinase-disintegrin TSV-DM (Contig_3958) were both expressed at higher levels. These contigs have the greatest sequence similarity to proteases identified in snake venom, shown to inhibit cell proliferation and platelet aggregation in cultured cells [43,44]. It is possible that these proteins in QPX contribute to similar inhibitory effects on *M* *. mercenaria* host cells. Two genes involved zinc transport were also expressed at elevated levels in warmer conditions: zinc transporter zupT (Contig_787), and zinc transporter ZIP12 (Contig_3533). The co-occurring upregulation of these genes with the former encoding zinc metalloproteinases supports the idea that these proteases may be virulence factors at higher temperatures. Further investigation to identify and quantify zinc metalloproteases in QPX and test their effects against *M* *. mercenaria* is necessary to understand their putative roles in QPX virulence.

Several genes associated with antibiotic biosynthesis were also found to contribute to QPX enriched biological processes. These genes were expressed at higher levels at the warmer incubation temperature, and include linear gramicidin synthase subunits B (Contig_8186), C (Contig_2578, Contig_5432, Contig_7823), and D (Contig_5631). Other QPX contigs associated with antibiotic biosynthesis include tyrocidine synthase 3 (Contig_9327) and surfactin synthase subunit 2 (Contig_9510). Tyrocidine and linear gramicidin work in concert to regulate the process of sporulation [45,46] and are associated with heat-tolerance in spores [45]. Moreover, gramicidin and tyrocidine are both associated with the release of extracellular proteases [46], and gramicidin D was found to be a potent molluscicide in zebra mussels [47]. In light of these findings, we postulate that these genes may serve several functions in QPX: to increase sporulation, coordinate release of virulence factors (i.e. proteases) and damage host tissue.

Previous studies have hypothesized that host thermal stress contributes to field observations of increased mortality in infected *M* *. mercenaria* at higher environmental temperatures [19]; however, upregulation of several potential virulence factors at higher experimental temperatures suggests that increased pathogen virulence may also play a role. Further investigation into thermotolerance and temperature-induced immunological changes to *M* *. mercenaria* , as well as production and function of potential virulence factors in QPX, is critical for quantifying the contribution of these two mechanisms to patterns of host mortality.

Reports of infectious diseases are increasing in commercial fisheries such as herring [48,49], salmon [50,51] and shellfish [52,53]. For many of these pathosystems, environmental parameters strongly influence the outcome (disease) by modulating host immune defenses and pathogen survival and virulence [16,54,55]. However, there is limited information on the mechanisms underlying these patterns, particularly at the molecular level. In this study, we analyzed the interaction of QPX with its environment through use of transcriptomic sequences from a pathogen cultured at different temperatures. These data were used both to establish a preliminary transcriptome and to identify potential mechanisms of virulence in QPX. The genomic characterization of QPX presented here will further contribute to a more thorough understanding of the pathogen’s biology and how the environment may influence it.

## Materials and Methods

### QPX Culture, Temperature Incubations and Sample Processing

QPX cultures used for this experiment were obtained from American Type Culture Collection (ATCC Number 50749; Manassas, VA) and maintained using culture conditions previously described [56]. Briefly, QPX was grown in MEM Eagle media in 25 cm^2^ culture flasks (21°C) and regularly transferred to new media. In order to assess the influence of incubation temperature on gene expression, QPX cultures were transferred to seawater and grown at either 10°C or 21°C for 72 hours. Specifically, excess mucus was removed to allow for accurate quantification of organisms and QPX was incubated at these two temperatures in sterile seawater (20 µL sterile seawater in 1 mL tube). Two tubes (1 mL) were used for each temperature treatment.

For RNA extractions, samples incubated at either 10°C or 21°C for 72 hours in seawater were pelleted and Tri-Reagent was added directly to cells for RNA isolation using the manufacturer’s protocol (Molecular Research Center, Inc., Cincinnati, OH). All samples were then DNased using the Ambion Turbo DNA-free protocol (Invitrogen, Grand Island, NY). Messenger RNA was isolated using the Ambion Micro Poly(A) Purist. In addition to RNA samples taken from QPX incubated at different temperatures, a separate 25 cm^2^ culture flask was processed to isolate DNA. Specifically, excess mucus and media was removed via centrifugation and genomic DNA was isolated using DNAzol (Molecular Research Center, Inc., Cincinnati, OH) according to manufacturer’s protocols.

### High Throughput Sequencing and Assembly

Three libraries were constructed and sequenced by the University of Washington High Throughput Genomics Unit at the University of Washington, using the Illumina HiSeq platform (San Diego, CA): two transcriptomic libraries (QPX incubated at 10°C and 21°C) and one genomic DNA library. Quality trimming of resulting sequencing reads was performed using CLC Genomics Workbench v. 5.0 (CLC Bio, Germany) with the following parameters: quality limit = 0.05 (Phred) [57,58]; number of ambiguous nucleotides < 2 on ends, and reads shorter than 25 bp were removed. The two transcriptomic libraries were assembled to generate a QPX transcriptome, while the genomic library reads were assembled to provide contigs representing the partial genome of QPX. *De novo* assembly was performed with Genomics Workbench v. 5.0 (CLC Bio, Germany) on quality trimmed sequences with the following parameters: mismatch cost = 2, deletion cost = 3, similarity fraction = 0.9, insertion cost = 3, length fraction = 0.8 and minimum contig size of 100 bp for genomic data and 200 bp for transcriptomic data. In order to remove ribosomal RNA sequences from the transcriptome data, consensus sequences were compared to the NCBI nt database using the BLASTn algorithm [59]. Sequences with significant matches (9) were removed and not considered in subsequent analyses.

### Genomic Sequence Annotation and SNP Detection

Contig sequences from the *de novo* assembly of the genomic library were annotated by comparing sequences to NCBI Nucleotide and UniProtKB/Swiss-Prot databases. Comparisons were made using the BLASTn and BLASTx [59] algorithms, respectively, with an e-value threshold of 1.0E-5. SNP detection was carried out on the genomic DNA assembly using the following parameters: mismatch count = 2, window length = 11, minimum average quality of surrounding bases = 15, minimum coverage = 25, minimum variant frequency = 35%. Putative SNPs located in the first 25 bp of the contig were excluded. Genomic feature tracks including SNP, RNA-Seq, and putative gene identification information corresponding to the 555 largest genomic contigs were developed and published [60].

### Transcriptome Annotation and SNP Detection

Transcriptomic sequences were annotated by comparing contiguous sequences to the UniProtKB/Swiss-Prot database [61]. Comparisons were made using the BLASTx algorithm [57] with a 1.0E-5 e-value threshold. Genes were classified according to Swiss-Prot Gene Ontology (GO) associations, as well as respective parent categories (GO Slim).

Transcriptome-based SNP detection was carried out on reads mapped to the contigs from the *de novo* assembly of the transcriptome. The following parameters were used: mismatch count = 2, window length = 11, minimum average quality of surrounding bases = 15, minimum coverage = 10, and minimum variant frequency = 35%, and maximum expected variations (ploidy) = 2 (CLC Genomics Workbench v. 5.0 (CLC Bio, Germany)).

### RNA-Seq Analysis

For RNA-Seq analysis, expression values were measured as RPKM (reads per exon model per million mapped reads) [62] with an unspecific match limit of 10 and maximum number of 2 mismatches (CLC Genomics Workbench v. 5.0 (CLC Bio, Germany)). Statistical comparison of RPKM values between the two libraries was carried out using Kal’s test [63], and multiple comparison correction was performed using a false discovery rate. Genes were considered differentially expressed in a given library when the p-value was less than or equal to 0.05.

### Gene Enrichment Analysis

Significantly enriched GO terms associated with differentially expressed genes were identified using the Database for Annotation, Visualization and Integrated Discovery (DAVID) v. 6.7 [64,65]. The specific GO category (GO FAT), developed as part of the Annotation Tool of the DAVID suite of bioinformatics resources, is a category that filters out very broad GO terms based on a measured specificity of each term. The UniProt accession numbers for differentially expressed genes were uploaded as a gene list, while all UniProt accession numbers for annotated transcriptomic contigs were used as a background. Enrichment analysis was performed separately based on relative expression directionality. Significantly enriched GO terms were identified as those with p < 0.05. Enriched GO terms were clustered in semantic space using Revigo [66].

## Supporting Information

Dataset S1 **QPX partial genome sequence (fasta file).**
(ZIP)

Dataset S2 **QPX transcriptome (fasta file).**
(ZIP)

Table S1 **Putative single nucleotide polymorphisms (SNPs) from genomic DNA sequencing.** 25,829 putative SNPs were identified across 7,734 DNA contigs. Data in the Table include Contig ID, Position of SNP (bp) within the contig, Base variation at the SNP (Allele Variation), Respective frequencies of alleles (Allele Frequencies), Allele Counts, Coverage at each loci (number of reads).
(TXT)

Table S2 **QPX transcriptome gene annotations.** Annotations correspond to nucleotide sequences available in Dataset S1 (QPX transcriptome) and include results from a sequence similarity searches using the SwissProt database. Data in the Table include Contig ID, SwissProt ID of top BLAST hit, Gene description of top BLAST hit, and corresponding e-value.
(TXT)

Table S3 **QPX transcriptome Gene Ontology (GO) information.** GO and GO Slim classifications provided for all annotated contigs. Data in the Table include Contig ID, SwissProt ID of top BLAST hit, Gene description of top BLAST hit, corresponding e-values, associated Gene Ontology (GO) term, associated GO domain, and associated GO Slim term.
(TXT)

Table S4 **Putative single nucleotide polymorphisms (SNPs) from transcriptome sequencing.** 1,925 putative SNPs were identified from 702 contigs in the de novo assembly of the transcriptome. Data in the Table include Contig ID, Position of SNP (bp) within the contig, Base variation at the SNP (Allele Variation), Respective frequencies of alleles (Allele Frequencies), Allele Counts, Coverage at each loci (number of reads).
(TXT)

Table S5 **Relative gene expression values (RPKM: reads per exon model per million mapped reads) between QPX cultures grown at 10C (QPX10) and 21C (QPX21).** Of the 11,271 contigs, 849 contigs in the QPX transcriptome were expressed at a higher level in the QPX10 library and 728 contigs were expressed at a higher level in the QPX21 library. Data in the Table include Feature ID, QPX10 RPKM values, QPX21 RPKM values, and results from Kal’s Z-test with FDR p-value correction. Genes were considered differentially expressed in a given library when the p-value was less than or equal to 0.05.
(TXT)

Table S6 **Gene enrichment analysis information on QPX10 and QPX21 libraries.** 26 biological processes were enriched that corresponded to 163 QPX transcriptome contigs in the QPX10 library; and 60 biological processes were enriched that corresponded to 175 QPX transcriptome contigs in the QPX21 library. Datasheets are provided separately for each library. Specific data in the Table include Gene Ontology (GO) category, GO Term (number and description), Enrichment p-value, Corresponding QPX Contig ID, SwissProt ID of top BLAST hit, Gene Description of top BLAST hit, and corresponding e-value associated with BLAST search.
(XLSX)
